# Higher Education in Public Health as a Tool to Reduce Disparities: Findings from an Exploratory Study among the Bedouin Community in Israel

**DOI:** 10.3390/ejihpe13100147

**Published:** 2023-09-30

**Authors:** Osnat Bashkin, Avia Suisa, Sharon Levi

**Affiliations:** Department of Public Health, Ashkelon Academic College, Ben-Tzvi 12, Ashkelon 78211, Israel; abihsois@edu.aac.ac.il (A.S.); sharonlevi@edu.aac.ac.il (S.L.)

**Keywords:** education, public health, academia, community, minority, bedouin population, disparities, internship

## Abstract

The Bedouin community is a minority disadvantaged population in Israel that suffers from a variety of health and socioeconomic disparities and limited access to higher education. The current study aimed to examine perceptions, successes, and challenges experienced by Bedouin students during their studies and to assess an internship program developed on the principles of a community-based participatory research approach to public health. In-depth interviews were conducted with 34 Bedouin students studying in the public health academic track between January and April 2023. Grounded Theory was used to analyze the data. Three main themes emerged from the analysis: (1) facilitators for the decision to pursue higher education in public health, (2) challenges and coping strategies, and (3) experiences of success. The internship program included eleven Bedouin students who conducted six community intervention projects covering a range of topics with different target Bedouin populations. Higher education is crucial for empowering minorities, producing leadership, and reducing socioeconomic and health gaps. The field internship enabled the necessary alignment between academia and public health practice. It is important to further reflect on the integration of minority groups in public health studies and its role in decreasing health inequity.

## 1. Introduction

The Bedouin population in southern Israel is one of the largest minority groups in Israeli society, constituting about 3% of the total population and 14% of the Arab population in Israel [1,2]. Residing in traditional and tribal villages, located in the periphery and characterized by a unique patriarchal social structure, the Bedouin community suffers from a variety of disparities as compared to other minority groups in Israel. The low socioeconomic and educational levels, limited availability of medical services, limitations of geographic mobility due to limited public transportation in the Bedouin villages, and language and socio-cultural barriers lead to difficulties in obtaining appropriate health services and thus contribute to significant health inequities [3,4,5,6]. 

There is an integral relationship between education and health within the structural and contextual frameworks of society, and it plays a fundamental role in the general well-being of individuals and societies [7]. Education affects health in various mediated links including improving employment and economic status [8], enhancing health behaviors [9], developing better social–psychological supportive circles in life [10], and improving access to healthcare [7]. 

### 1.1. Health Disparities among the Bedouin Population in Israel

It is widely acknowledged that health disparities are highly prevalent in Bedouin villages due to poor living conditions as well as cultural and socioeconomic factors. About half of the Bedouin population lives in unrecognized villages with poor sanitation and limited basic infrastructures such as electricity, water supply, and access to healthcare services [11]. As a result, poor health outcomes are common among the Bedouin population, including a high rate of congenital disorders due to consanguineous marriages [12,13], a high rate of infant mortality [14], an increasing rate of type 2 diabetes mellitus causing low life expectancy compared to Israeli Jews [15], a higher level of psychological distress [16], a predominance of injuries among Bedouin males [17], and more. 

There are various efforts to decrease health disparities and increase health equity for the Bedouin population in Israel, primarily employed by the Ministry of Health, health maintenance organizations, and non-governmental organizations in Israel. Among these efforts are the expansion of mother and child clinics in the Bedouin villages, health promotion workshops for Bedouin women, and training nurses and other health professionals in the Bedouin population, such as nurse case managers for diabetes Bedouin patients [18]. Despite these efforts, additional steps are necessary to significantly improve health outcomes, including using culturally sensitive, health-focused continuous interventions. 

### 1.2. Higher Education among the Bedouin Population in Israel

Higher education is important for empowering minority development, expanding the leadership required for socioeconomic development, and reducing health gaps [19]. However, evidence shows that the Bedouin population’s access to higher education is limited and challenging. There are several barriers to higher education among the Bedouin population; of significance is poor education services and a lack of resources at the pre-university stage. This includes, among other things, insufficient guidance for choosing an academic track. Due to this, many young Bedouins tend to choose the same study tracks, primarily academic programs in teaching and social sciences, although many of them prefer health sciences and medical programs. Therefore, they are less likely to complete their university degrees [19,20]. Additional barriers to higher education among the Bedouin population include the admissions procedures in Israeli universities, which are challenging for young Bedouins [21], language barriers, the lack of sufficient financial resources for Bedouins for university studies, cultural and traditional gender roles, inadequate financial resources, and physical access barriers [22]. 

One of the latest intervention programs to decrease inequity in higher education among the Bedouin population is called “Gateway to Academia” developed and implemented by the Council of Higher Education in Israel as of 2016. The program assists Bedouins to integrate into higher education studies, even if they do not meet admissions requirements, by providing a one-year program (pre-undergraduate studies) provided in small group classes focusing on extra English and Hebrew language training and academic literacy, alongside financial and social support [23,24]. Recent assessments of the program outcomes reveal high cognitive, economic, emotional, and social satisfaction among Bedouin students participating in the program [25]. 

### 1.3. Integrating Bedouins in Public Health 

As part of the “Gateway to Academia” program, as of 2019, young Bedouins were integrated into an undergraduate degree in public health at the Ashkelon Academic College (AAC). The undergraduate public health track was established in 2014 at AAC, located in southern Israel. The process, challenges, and achievements of this unique public health track are described in a previous article [26].

The Bedouin students enrolled in the “Gateway to Academia” program at AAC are provided with academic skills, social and financial support, and a few basic introductory courses in health sciences. At the end of the pre-undergraduate one-year program, the students choose one of the three academic tracks included in the AAC School of Health Sciences: Nutrition, Nursing, and Public Health. To date, 34 Bedouin students study in the Public Health undergraduate track. To assist the Bedouin students in successfully integrating into the undergraduate public health track, we implemented several tools including periodic personal follow-up meetings of academic staff with Bedouin students, strengthening the social relationships between Jewish and Bedouin students through various activities, mentoring programs, personal tutoring by graduate students, and providing additional practice hours for Bedouins students. Moreover, in 2022–23, an internship program was developed and implemented with the participation of Bedouin students to integrate public health students in the field and reduce health disparities in their communities.

At the end of four years of participation in the public health track, an understanding of the integration of Bedouin students is important for future planning and expansion. Therefore, the current study aimed to examine perceptions, needs, successes, and challenges experienced by Bedouin students during their studies. In addition, we describe the implementation of an internship program developed on the principles of a community-based participatory research approach to public health to mitigate health and education disparities among the Bedouin community. Both the interviews and the internship program lay the groundwork for the future development of interventions to alleviate gaps in education and health among minority groups. 

## 2. Materials and Methods

This exploratory study was conducted to gain in-depth insights into how Bedouin students perceive their experiences in the public health academic track. The research includes qualitative in-depth interviews and a review of intervention projects implemented by Bedouin students in their communities. The Consolidated Criteria for Reporting Qualitative Research (COREQ) checklist was used to report the study [27]. The study was approved by the Ashkelon Academic College Ethics Committee (Approval # 57-2023). 

### 2.1. Interview Participants

Interview participants were recruited using purposive sampling. In purposive sampling methods, researchers select individuals who meet specific prescribed criteria [28]. The criteria was being a Bedouin student in the Public Health undergraduate track. All 34 Bedouin students studying in the public health academic track at the AAC were recruited for the study and were interviewed between January and April 2023. Interviewees included 10 men and 24 women; 9 of them were first-year students, 10 were second-year students, and 15 were third-year students. The Bedouin students completed matriculation in local high schools. While the high schools in the Bedouin communities often have poorer education services, the students all had the opportunity to participate in the “Gateway to Academia” program at AAC in order to reduce gaps and allow them to qualify according to the same admission standards as students from other backgrounds. In the public health track, all of the Bedouin students are at the 35th percentile of scores. The students’ age ranged between 19 and 23. Participants gave informed consent for inclusion in the study and were informed about the methods to protect the data for anonymity and privacy.

### 2.2. Qualitative Data Collection 

Data were collected using a semi-structured interview format. The topics considered in the development of the interview guide included the decision to pursue higher education and to study public health, challenges, and unmet needs during their studies, sources for support, and successful experiences. 

The interview guide was comprised of non-directive and open-ended questions about perceptions and experiences during academic studies (Appendix A). The wording and order of the questions were adapted according to the interview dynamics to maintain continuity and flow and encourage openness of interviewees. The content validation method was used to ensure that the questions in the guide were relevant to the study goals. The guide was pilot tested with two students to ensure a smooth interview flow and verify comprehension of the questions.

The interviewer was a third-year student in public health, trained in qualitative research methods, and supervised by the corresponding authors. All interviews were conducted face to face, individually, at the college and lasted 30–60 min. Interviews were audiotaped and transcribed verbatim in Hebrew in a standardized format. It was emphasized to all interviewees that their details would remain confidential, that they did not have to answer all the questions, and that they could stop the interview at any time. In addition, all interviewees approved the recording and transcript of their interview.

### 2.3. Qualitative Data Analysis

Interview transcripts were analyzed by the authors using a thematic analysis method based on Grounded Theory [29]. Interpretive analysis was performed soon after the interviews were conducted. The analysis included incorporating deductive and inductive themes that arose from the research topics based on the literature and from the research data [30]. The analysis stages included: (1) a literal reading of all the interviews to gain a comprehensive picture of the data; (2) identifying categories and themes related to the research objectives; (3) redefining central themes to include encoded quotes and examples based on re-reading the transcripts; (4) the themes and quotes were translated and documented in English. An ongoing internal quality audit was conducted to determine whether the data were collected, analyzed, and reported consistently following the study protocol [30]. 

### 2.4. Community Intervention Projects

In parallel to the qualitative interviews, a special internship program was piloted for third-year students. The purpose of the internship was to utilize academic skills acquired during public health track studies to reduce health disparities in the community. The internship was coordinated and guided by the authors and lasted eight months. The internship program was developed on the principles of the community-based participatory research (CBPR) approach to public health, a collaborative research approach that involves active participation of community members, researchers, and other stakeholders [31]. The CBPR method has long been recognized as a method to cope with health disparities [32]. In CBPR, the partnership between researchers and community members is emphasized with the goal of addressing issues and concerns that are relevant and meaningful to the community being studied, as intended when involving Bedouin students in actions that facilitate a change in the lifestyle and behaviors within their communities. As action researchers and public health representatives within their community, the Bedouin students are better able to question and grapple with issues that other researchers or professionals may be able to access as well as translate knowledge for the target population [33]. 

The internship program included three phases:

1. The first stage included identifying a public health issue in the Bedouin community that needs to be addressed and conducting a literature review.
2. The second stage included the development of small-scale health-promotion interventions aimed at addressing the defined problem in their community using models, tools, and competencies taught and acquired during their studies. The students developed materials, mentored by the course instructors. The students were required to work with partners in the community and seek and gain collaboration from different organizations to finalize and conduct the intervention project that they developed.
3. The third stage included the implementation of their intervention. The students were responsible for presenting the sessions and leading the discussions in the community. The student conducted a process and outcomes assessment, which was presented at the end of the year in the college, as well as community and personal reflection.

The internship program included 11 students who conducted 6 community intervention projects. The program was assessed for its contribution to the integration of Bedouin students in the public health field, feedback on the effects of the projects in the community, and the influence of the projects on the students’ perceptions and experiences.

## 3. Results

The interviews revealed common perceptions and experiences across the Bedouin student population. Analysis of the data from the interviews revealed three main themes: (1) facilitators for the decision to pursue higher education in public health, (2) challenges and coping strategies, and (3) experiences of success.

### 3.1. Theme 1: Facilitators for the Decision to Pursue Higher Education in Public Health

Most of the interviewees described the decision to pursue higher education as one that was made with significant encouragement from family members or high school teachers. Many students described the poor employment of their parents as a major factor affecting their decision to participate in higher education and the “Gateway to Academia” program. Most of the interviewees see the program as a significant tool helping them integrate into academic studies and appreciate the opportunity they have received to be better prepared for academia. They mentioned the challenge of meeting the requirements needed for enrolling in universities in Israel, in particular the psychometric test, which is mandatory for most of the academic tracks. 

Many indicated their desire to study a field that could improve the health of their community as a main factor in the decision to study public health. Some students have relatives or other important role models who work in the health field or inspired them to enroll in the public health track. 

Female student, 20 years old, second year:

*The decision to study public health was not my decision but my teacher’s. He told me that there is a field that he thought would suit me, I wasn’t familiar with this profession and the teacher just pushed me into it. I discovered that I really like this field, the studies and the field are very interesting, and I was really attracted to it. I decided that I wanted to do a master’s degree in public health because it is more interesting to me and important in my community.*

It is important to note that many of the interviewees described their former plan to study nursing but since they did not meet the admissions requirements, they decided to study in the public health track and attend an accelerated track for academics to transition to nursing in the future.

Male student, 19 years old, first year:

*I came here at first because you can do an accelerated transition to nursing at the college after graduating in public health. My matriculation grades were high, and I wanted to study nursing because that’s what I relate to the most. I took psychometrics several times, but I could not get a satisfactory score. But now things have changed, I want to do a master’s degree in public health, I like the field.*

Female student, 23 years old, second year:

*From a young age, I wanted and aspired to get into a higher education institution to study something related to medicine. I chose to study public health because it was the only way to get into nursing without the psychometric exam. I focus on moving on, telling myself that I will succeed because it won’t help me to complain and stand still. I need to move forward. Everyone helps me, especially my parents.*

### 3.2. Theme 2: Challenges and Coping Strategies

The geographical distance and mobility barriers were mentioned by most of the students. As mentioned above, the Bedouin community suffers from insufficient internal and external public transportation networks, which affect students’ mobility and access to the campus located about 60 km from the main Bedouin town and 80 km from most of the Bedouin villages. The support they receive from parents was mentioned as an important factor in coping with this barrier. 

Male student, 19 years old, first year:

*Coming here from my home is the most challenging aspect for me. Despite the distance, I wake up at five in the morning and make my way here. My father is one of the people who helps me out; every day he expresses his pride in me and wishes to see me succeed.*

Female student, 22 years old, third year:

*My most significant difficulty all these years is that I come from far away to study here, and I must take so many bus trips that are hard for me. I leave the house really early in the morning and ask Allah to help me stay strong and finish this degree.*

Many of the participants stated that the language barrier significantly affects their ability to succeed. The language barrier often forced the Bedouin students to translate the learning material and lecturers into Arabic-Bedouin, causing them excessive workload and stress during their studies. Some have referred to the willingness of relatives and family members to assist in translation as a significant factor in their academic success. Others mentioned the assistance of their classmates and stated that forming close relationships with Hebrew-speaking classmates helped them better understand the language and the learning material. 

Female student, 23 years old, third year:

*The first year was very difficult for me, I did not know how to speak Hebrew well, and the language was very difficult for me. The hardest thing for me was the difficulty in expressing myself in class and also doing presentations in front of the class, it really challenged me. During the years I tried to explain myself to the Jewish students, the lecturers tried to understand me, and I tried to understand them and that’s how it works out. I got used to it and learned to accept it.*

The students mentioned limited cultural competency in the academic system and among some of the academic staff as a factor that poses a challenge for them, especially in situations of asking for help from the academic staff. Female Bedouin students emphasized the continuous need to explain gender-based cultural challenges they face during their studies. For example, due to the socio-cultural structure of the Bedouin society and gender-based norms, Bedouin women feel embarrassment that prevents them from speaking in class during presentations or catching a ride from the village to the campus with a male student. In addition, several of the female Bedouin students are married and pregnant as expected from a woman their age in Bedouin society, which adds difficulties to maintaining a study routine and academic continuity. Nevertheless, the feelings of being a role model for women in the community and a sense of mission to promote the health of the Bedouin population were common among female Bedouin interviewees. 

Female student, 22 years old, second year:

*Socially it’s not easy for me. Academically, in the beginning, it was hard, but the lecturers provided us with extra classes, and I see how I’m improving little by little. Without a degree, I am worth nothing, so it is important for me to succeed. The hardest part is presenting in front of the class, it scares me the most. I practice at home in front of my family, they correct me when necessary and advise me how to speak. They give me a lot of confidence and tell me not to be afraid and that I will succeed in the end. I want to study for an advanced degree in physical activity because it is lacking in the Bedouin community.*

Female student, 23 years old, second year:

*I decided to go to higher education because I think that the Bedouin society needs to progress, Bedouin women are not sufficiently educated, and I decided to be one of those who are. Public health is a field of knowledge that I really like. I am less connected to the practice of medicine on an individual level, but more on a community level, and I think that this thing is lacking in our community, that’s why I chose public health.*

Female student, 23 years old, third year:

*According to my perception, every woman should have a bachelor’s degree because I see that when a woman studies, her thinking changes. But it was difficult for me that people here at the academy did not understand our culture and our difficulties. Many times, I wanted to quit my studies and then I saw how far I had come, how much I had achieved. I continued my study, and it makes me the happiest. This is my greatest success.*

### 3.3. Theme 3: Experiences of Success

Overall, the students voiced experiences of success alongside the different challenges they face, in particular as they progress through their academic studies. All the interviewees mentioned good grades and the ability to continue to advance academic studies as the main success. Some of the first- and second-year students stated the ability to form good relationships with their Jewish peers and speak Hebrew fluently as an important accomplishment. 

Female student, 20 years old, second year:

*Some Jewish students like to talk to us, they mainly want to know about our culture, about our religion, so they ask, and we answer. It connects us and helps us to feel comfortable in class. In addition, it is very helpful to get assistance from Bedouin students in advanced years who give smart tips and advice. My most successful and significant experience in this degree is that I manage to connect with people and cope successfully with challenges in my studies.*

Third-year students specifically felt that they succeeded in the challenging journey of academic studies and described feelings of satisfaction, pride, and empowerment. Most of them stated that they feel self-confident in their academic and social abilities and their capability to cope with the academic challenges they faced during their studies. All of the Third-year students stated that they feel confident in their ability to contribute to and promote Bedouin community health. 

Male student, 24 years old, third year:

*I started academic studies on a different track at first. After a while, I decided to switch to the public health track. I realized that the issues of public health really interest me. I have an attention disorder, so it was very difficult for me. I searched and found methods to cope with the difficulty. I recorded the lectures and used other Jewish students’ summaries in class. At home, I invested time in understanding the learning material and I succeeded. My academic achievements are high, and I am proud of myself for that. This is my biggest success, and it encourages me to pursue a master’s degree in public health.*

### 3.4. Community Intervention Experiences in the Bedouin Community

During the internship, students experienced challenges and successes, implementing academic competencies, public health knowledge, and leadership as well as accountability skills in practice. The internship community projects covered a range of topics with different target populations (see Table 1). These topics were all selected based on the identification of an issue demanding action research and intervention within the Bedouin community. 

All of the projects were coordinated with organizations in the community including schools, health organizations, municipal agencies, and NGOs. The projects were conducted in accordance with cultural norms as regards language, dress, and settings for example male public health students conducted meetings with men at community mosques; female public health students conducted physical activity promotion with adolescent girls in school.

In a review of the internship program and the community projects, several key findings emerge at the community level:

- There is a lack of programs engaged in knowledge transfer in the field of health for members of the Bedouin community;
- Serious knowledge gaps exist in all of the health topics that were covered in the various community programs;
- Cultural norms are often a barrier to health-promoting behaviors;
- Bedouin children, adolescents, and adults voiced a desire for tailored information and programming by members of the community;
- Intervention programs that align with cultural norms have the potential to reach a larger audience.

In several cases, partners in the community project requested additional programming and recommended that the projects continue in some format. Program participants provided positive feedback and evaluation results were promising.

A review of the internship program also sheds light on the influence of the projects on the students’ perceptions and experiences, including:

- Feelings of success, empowerment, and recognition of their vocation in increasing awareness and promotion of healthy behaviors within the community;
- Identification of knowledge gaps and cultural norms that may prevent the adoption of healthy behaviors within their community, particularly regarding gender disparities in the Bedouin community; for example, during recruitment for workshops with women, public health students met resistance from Bedouin husbands who would not permit their wives to attend;
- A desire to work within their community to continue to promote public health.

The internship program provided the Bedouin public health students with deeper insights into health determinants within their community and practical exposure to health promotion.

## 4. Discussion

The current study examined perceptions, needs, successes, and challenges experienced by Bedouin students during their studies and assessed an academic framework for implementing a field internship, including community intervention experiences, in the Bedouin community, as a tool to decrease academic and health disparities. Our findings help lay the groundwork for the future development of community-based interventions to alleviate education and health gaps among minority groups. 

The interviews revealed common perceptions and experiences across the Bedouin student population. The main findings indicate that young Bedouins experience a major challenge in meeting the requirements needed for enrolling in universities in Israel and see the “Gateway to Academia” program as a valuable opportunity to help them integrate into higher education. This finding is in line with previous studies describing the admission procedures to higher education institutions as a significant obstacle to minority and disadvantaged populations acquiring higher education [21]. Admission procedures including psychometric exams are not good predictors of academic abilities among minority students, causing a culturally based disadvantage for students who wish to enroll in higher education institutions [34]. In this context, a structured population-tailored solution such as that offered by the “Gateway to Academia” program is valuable in assisting minority students to cross this deep socio-cultural barrier and improving the access and integration of the Bedouin community to higher education. 

Several major challenges were mentioned by the Bedouin students: geographical distance and mobility barriers, language barriers, and cultural and gender-based barriers. These challenges mentioned in previous studies conducted among minorities and the Bedouin population need to be carefully addressed [19,22]. Since educational attainment is one of the key factors affecting the Bedouin community’s socioeconomic status, it is important to develop innovative plans to promote education and professional development while addressing these social and structural barriers through necessary changes in government policy. Further consideration of appropriate additional support for Bedouin students, in particular during the first and most challenging year of academia, is recommended. Moreover, additional training on cultural competency for academic staff is recommended to improve their understanding and recognition of these aforementioned gaps. Cultural competency training of faculty and trainers in the health field is recognized as imperative to support a culturally diverse patient population [35]. Moreover, in public health, cultural competency training is recognized as essential to target population-based health and reduce health disparities, including culturally sensitive research, programming, and evaluation [36]. 

Along with the barriers mentioned, our students noted experiences of success in the challenging journey of academic studies and described feelings of satisfaction, pride, and empowerment. Considering most of them indicated their initial desire to study a field that could improve the health of their community as a main factor in the decision to study public health, it was encouraging to see that all of the third-year students stated that they feel confident in their ability to contribute to and promote Bedouin community health. These findings were further supported by their positive experiences conducting small-scale intervention projects in the community during the internship program. The beliefs and attitudes regarding increased self-efficacy that the Bedouin students developed over the course of their studies have the potential to contribute significantly to their future performance in the fields of public health practice and research [37,38].

Based on our experience implementing field internships in the Bedouin communities, higher education is crucial for empowering minorities, producing the leadership necessary for social and economic progress, and reducing socioeconomic and health gaps among minority groups. Moreover, the field internship enabled the necessary alignment between academia and public health practice, which is known to improve public health actions, by conducting population-adjusted and collaborative interventions for complex public health issues [39]. Involving Bedouin public health students in community-based participatory research and programming within their community adds the element of culture-centeredness, which is an important strategy in confronting social determinants of health and reducing health disparities [40]. Accordingly, the Bedouin students who participated in the internship had the opportunity to explore and gain an in-depth understanding of the ways to deal with the variety of health determinants in their community, initiating collaborations and obtaining leadership competencies that impact the lived experiences and realities of their community [41,42]. Finally, the community-tailored field internship offered undergraduate students exposure to potential employment opportunities after graduation, which may fulfill the need for highly qualified and skilled public health professionals with the appropriate foundation, practical experience, and knowledge of specific community structures [43,44].

This study has several limitations. First, the study was conducted in Israel, among a single minority group, the Bedouin community; therefore, it may be difficult to generalize the findings to other countries. In addition, we did not compare the experiences reported by the Bedouin students to those of other cultural groups of students. It is possible that such a comparison would have revealed additional organizational and social factors that may influence the students’ experiences, difficulties, and successes. Nevertheless, we believe that our methodology, which included both qualitative interviews and the internship development and assessment, enables replication in other academic programs enrolling minorities aiming to decrease disparities among minority groups.

## 5. Conclusions

The integration of Bedouin students in academic studies is complex and challenging. It is important to reflect on the complete picture regarding the integration of Bedouin students in public health studies to adapt and meet the unique needs that were identified in this study. The findings of the research reveal the specific challenges and barriers students face in public health studies, as well as their achievements and successes, and enable a deeper understanding of the Bedouin student’s perceptions and experiences. We believe that our experience with the integration strategies and the community internship model may be replicated in other academic programs aimed at decreasing disparities among minority groups.

## Figures and Tables

**Table 1:** Overview of internship community projects in the Bedouin community.

Topic	Target Population	Intervention Design	Quotes from Participants/Key Findings
Prevention of congenital disorders	Women in childbirth years	Two sessions were conducted with the welfare department (N = 30); information sharing and discussions regarding the causes of congenital disorders and the importance of early screening.	I always thought that disorders/diseases were my fault, but I learned in this meeting that the responsibility is shared between my husband and myself.
Prevention of early childbirth	Women with a high-risk pregnancy	Qualitative focus group sessions (N = 7) which included discussions and provision of information.	There is a lack of workshops on childbirth… This is the first time the clinic invited us to participate in this type of program.
Promotion of healthy nutrition	Adolescent girls (15–16 years old)	Four sessions in a high school setting (N = 36) provision of information on healthy nutrition and body image; short pre-post survey.	During the program due to the success, there was a request for additional meetings. Adolescents reported higher rates of healthy nutrition.
Promotion of physical activity (PA)	Adolescent girls (15 years old)	Three sessions in two schools including the provision of information, PA sessions with an instructor, and a short pre-post survey; a school with a designated PA room for girls (N = 15) and a school without a designated PA (N = 15)	In schools with a PA room, there were higher PA rates in school; following sessions, both groups reported higher PA rates outside of school hours and a higher rate of in-school PA rates also in the school without a designated PA room. A joint WhatsApp group was developed to share positive feedback and promote social norms.
Smoking prevention	Adolescent boys and girls (13–14 years)	Three sessions in a middle school setting (N = 30), including provision of information, demonstration of effects of smoking, and short pre-post survey	Adolescents spoke about problematic norms, in the family, among peers, and in the community. My father prepares at least 10 nargila (hookah water pipe) heads for guests that visit over the weekend. We always buy cigarettes at the neighborhood shop and the owner doesn’t even ask us who they are for (despite regulations).
Prevention of child unintentional injuries (backover crashes)	Men with children aged 0–4	Two sessions a month apart in the mosque (in multiple groups, total N = 50), provision of information, demonstration of the field of vision while backing up, and short pre-post survey	Significant increases in reported knowledge regarding distance in the field of view, behavior checking behind the vehicle prior to backup, and environment setting up a separation between the vehicle and play areas.

## Data Availability

The data supporting this study’s findings are available on request from the corresponding author. The data are not publicly available due to privacy or ethical restrictions.

## Appendix A

Semi-Structured Interview Guide:

The following interview is designed to learn from you about your experiences as a public health student here at Ashkelon Academic College. The interview is anonymous, your participation is completely voluntary, and you can request to stop the interview at any time.

1. Tell me a bit about yourself, how old are you and what year of academic study are you in?
2. Please share with me how you came to the decision to study in academia (institute for higher learning)?
3. Why did you decide to study your undergraduate degree in Public Health?
4. How would you describe your acclimation to academic studies? (Probe: Would you describe it as successful?)
5. Tell me a bit about any challenges you faced in your studies? (Probe: What would you say is the main difficulty or challenge)
6. (If there were challenges) How did you cope with the challenges? (Probe: What or who helped you?)
7. Do you feel you have support from your family?
8. Do you have any needs or requests that have not been resolved with the staff at the college?
9. Are you considering continuing for further academic or graduate studies at the end of this program?
10. Please share with me any successful experiences you had during your studies. (Probe: What would you say was the main successful experience? What helped you to be successful?)
